# Considering Objective and Subjective Measures for Police Use of Force Evaluation

**DOI:** 10.3390/ijerph18105351

**Published:** 2021-05-18

**Authors:** Paula M. Di Nota, Jennifer F. Chan, Juha-Matti Huhta, Judith P. Andersen

**Affiliations:** 1 Department of Psychology, University of Toronto Mississauga, Mississauga, ON L5L 1C6, Canada; paula.dinota@utoronto.ca (P.M.D.N.); jenniferf.chan@utoronto.ca (J.F.C.); 2 Police University College, 33721 Tampere, Finland; juha-matti.huhta@poliisi.fi; 3 Faculty of Education and Culture, University of Tampere, 33100 Tampere, Finland

**Keywords:** evaluation, assessment, objective measures, subjective measures, decision-making, situation awareness, police, use of force

## Abstract

In spite of significant interest in the application of police use of force (UOF) from organisations, researchers, and the general public, there remains no industry standard for how police UOF is trained, and by extension, evaluated. While certain UOF behaviours can be objectively measured (e.g., correct shoot/no shoot decision making (DM), shot accuracy), the subjective evaluation of many UOF skills (e.g., situation awareness, SA) falls to the discretion of individual instructors. The aim of the current brief communication is to consider the operationalisation of essential UOF behaviours as objective and subjective measures, respectively. Using longitudinal data from a sample of Canadian police officers (*n* = 57) evaluated during UOF training scenarios, we discuss how objective and subjective measures reflect changes in officer performance over time. Objective lethal force DM was measured as a binary ‘correct–incorrect’ outcome and subjective SA was measured on a 5-point Likert scale ranging from ‘unacceptable’ to ‘exceptional’. Subjective evaluation of SA demonstrated significant changes over time, while DM remained relatively high and stable. Given the practical and professional implications of UOF, we recommend that a combination of objective and subjective measures is systematically implemented at all stages of police UOF training and evaluation (i.e., basic, advanced, in-service).

## 1. Introduction

Police use of force (UOF) has received significant attention in recent years, including calls to revise existing training and practices by various stakeholders [1,2]. The extensive psychological impact of UOF encounters, especially those involving the use of lethal force, include operational stress injuries (OSIs) for police [3] and incalculable human suffering. Despite the gravity of UOF outcomes on both police and the public, there is currently no industry-wide standard for how police UOF is defined or trained [4,5]. By extension, there is no current standard for how essential skills, competencies, and behaviours related to UOF are evaluated. Given that UOF evaluations determine an officer’s fitness for duty (i.e., qualification and certification), empirical evaluation of current practices is necessary to ensure that necessary skills and competencies are adequately measured and reported. 

Police performance is typically reported as organisational or community-related indicators such as arrest rates and response times (see [6] for review). For the purpose of the current communication, we define UOF performance as specific operational behaviours and cognitive competencies related to the selection and use of various force options, including situation awareness (SA) and lethal force decision making (DM) [4,7]. Existing definitions of police UOF behaviour can be gleaned from applied research on training [8,9,10,11]. However, outcomes tend to be reductionistic (e.g., correct or incorrect ‘shoot/no-shoot’ DM or global ratings of SA) for ease of administration and interpretation [12]. Evaluations of psychological competency are typically based on pre-employment personality assessments, which have little to no connection to operational UOF performance in stressful contexts [13]. 

### Operationalising Police Performance for Evaluation Purposes

Only recently have researchers directly addressed the need to expand, clarify, and standardise UOF evaluation measures and procedures. Notably, these researchers are also experienced practitioners and instructors in police tactics and UOF. Using a systematic protocol, Bertilsson et al. [14] operationalises several perceptual, cognitive, and motor skills related to UOF performance in experienced officers. Outcome measures include verbal content, spatial and tactical implementation, and control of voice, movement, and the overall situation. Rooted in Endsley’s [15,16] definition and measurement of SA, Huhta and colleagues [17] dissect SA as a process that requires perception and understanding of the environment to predict and inform subsequent action. SA is operationalised as a set of implicit observable behaviours exhibited by novices and experts alike, including operational flexibility, initiative, and withdrawal and target-oriented behaviours. Preliminary analyses of the connection between Huhta’s behavioural dimensions and personality traits also provide insights on how to pedagogically tailor UOF and SA instruction. Most relevant for the current study, Koedijk et al. [12] propose a comprehensive methodological approach to the assessment of psychological and behavioural competencies in police. The authors provide an illustrative example of UOF evaluation that can be modified for a variety of operational contexts. Consideration is given to several stages of evaluation development and implementation, including identifying the basis of the test measure (i.e., intended competencies and behaviours), designing representative assessments (i.e., scenarios or tasks) that will elicit directly observable behaviours, as well as considering scoring method and interpretation. 

Despite these important contributions, UOF performance outcomes are typically rated on simplistic objective or subjective scales. Instructor ratings can be based on direct behavioural observation and/or consideration of officer explanations during or following a scenario (e.g., “What did you see?”, “How do you feel you performed?”). Objective measures such as the presence or absence of behaviour (e.g., shoot/no-shoot) are often more clearly operationalised, making them less ambiguous and susceptible to evaluator bias than subjective measures. Additional objective rating scales (where relevant) can include frequency of behaviour (e.g., number of shots fired), accuracy (e.g., distance from target), or completion time. Subjective measures including the behavioural competencies defined above [14,17] are typically scored on scales that can vary in type (e.g., Likert, visual analog), range (e.g., discrete points, or marking and measuring distance on a line), and criteria for each point on the scale (e.g., undefined range between ‘unacceptable’ to ‘exceptional’; specific points such as ‘never’, ‘sometimes’, ‘often’, ‘always’). Five- to 7-point scales have shown better reliability, validity, and discriminatory power than scales with fewer response categories, and 7- to 10-point scales are preferred by respondents [18]. 

While subjective measures can afford more detailed data than objective measures (i.e., the extent of competence), they are more prone to evaluator biases including the instructor’s own skill level and past experiences in training, instruction, or in the field [19]. Evaluator ratings can also be biased by what Koedijk et al. [12] refer to as performance considerations (i.e., evaluators’ abilities in observing, recognising, and interpreting behaviours of interest), conceptual considerations (i.e., susceptibility to bias from interpersonal relations, amount of required inference), and practical considerations (e.g., availability and cost of evaluators, pressure from management to favourably evaluate all officers in a short amount of time) [20]. Accordingly, global ‘pass/fail’ scores are often confused as objective measures, despite the fact that they require significant interpretation of an officer’s competence on any number of individual psychological and behavioural competencies. Methodological decisions surrounding how UOF performance is evaluated are further complicated, given that an officer’s fitness for duty cannot be represented by a single behaviour, and that fitness criteria are inconsistently defined by police organisations or legislation. There is a dearth of research directly comparing the benefits and limitations of subjective and objective measures of police UOF performance, and leaves the following questions unanswered: How consistently do objective and subjective measures represent overall competency in UOF performance? Do objective and subjective UOF performance measures reflect similar changes to competency over time? 

The goal of the present communication is to descriptively consider whether objective and subjective measures of police UOF performance consistently reflect competency over time. To exemplify our discussion, we use existing data from a longitudinal within-subjects field analysis of a police training intervention [9] to compare patterns of subjective measures of SA and objective measures of lethal force DM over the course of 18 months. If objective and subjective measures both reliably reflect overall competency in UOF performance, then subjective SA scores are expected to reflect similar patterns to objective DM scores across evaluations.

## 2. Materials and Methods

For more detailed materials and methods for the example data presented below, please refer to [9].

### 2.1. Participants

A total of fifty-seven (*n* = 57, 7 female) active-duty frontline officers employed by a large Canadian municipal police agency participated in this study (*M_Age_* = 32.8, *SD_Age_* = 6.3). Participants provided informed consent prior to volunteering for the study and were informed they were eligible to withdraw from the study at any point without consequence. All participants completed evaluations before and after a 4-day resilience and performance intervention. At least 49% of participants returned at each follow-up evaluation conducted 6-, 12-, and 18-months post-training, with 80.7% returning for at least one follow-up evaluation (see Table 1 for details). All procedures were approved by the University of Toronto Research Ethics Committee.

### 2.2. Procedure

Officers were evaluated at five time points: pre- and post-training, and at 6-, 12-, and 18-month follow-up evaluations. On the pre-training evaluation day, officers completed four live-action reality-based scenarios (see description below), one of which was specifically designed to test verbal communication and interpersonal skills (i.e., building rapport) and was excluded from analyses. Following the morning of pre-training evaluation scenarios, the intervention followed with two days of psychoeducational and scenario-based instruction. Day 4 consisted of the post-training evaluation, which was a single extended scenario that required three lethal force decisions. Evaluations at 6-, 12-, and 18-month follow-ups each consisted of three lethal force scenarios with one lethal force decision each. See Table 1 for the full evaluation schedule.

### 2.3. Reality-Based Evaluation Scenarios

Scenarios were designed by expert UOF instructors with over 10 years of occupational experience creating reality-based critical incident scenarios for training and evaluation purposes. As they were independent from the research team, the instructors designed scenarios that were both challenging and representative of what officers would typically encounter in the field at that agency (i.e., increasing ecological validity and consistency in scenario difficulty). The content of scenarios during training and evaluation included call responses such as domestic disturbances, a robbery in progress, attending to an individual in crisis, potential suicide, and an active school shooting (post-training scenario). Scenarios were conducted at an empty school that allowed for both indoor and outdoor environments (e.g., vehicle stops, staged apartments, classrooms), and props were used to create realistic scenes (e.g., fake blood, simulated weapons, scene-relevant attire, and furniture). Officers were fitted with training versions of their usual police equipment (e.g., vest, baton, conducted electrical weapon, duty weapon, and OC spray) and were exposed to the same scenarios but not in the same order. Consistency in scenario delivery was achieved by holding practice sessions for the actors at the location of the study prior to data collection, assuring systematic interactions across all scenarios for all officers.

### 2.4. Measures

Each participant was scored on their lethal force DM and SA performance by qualified UOF instructors independent from the research team and who did not design the scenarios. Each scenario was facilitated by a single UOF instructor, who provided verbal feedback and completed DM and SA scores for each officer immediately following the completion of the scenario. Participants were not made aware of their DM or SA scores, which were collected for research purposes only (i.e., to measure the effectiveness of a resilience training program, see [9]). 

Objective Lethal Force DM—To maintain equivalency in the number of lethal force decisions across time points, officers performed three scenarios at each evaluation with a single lethal force decision each, with the exception of the single post-training extended scenario that had three lethal force decisions. Scenarios were closely balanced in the type of lethal force decision required, which are defined as follows:

Shoot: using lethal force when appropriate situational criteria have been met.

No-Shoot: withholding the use of lethal force when appropriate situational criteria have been met (e.g., suspect complies with officer instructions). 

Officers were scored on a binary scale (1—correct; 0—incorrect) for each lethal force decision. For each evaluation, binary DM scores were summed across scenarios (out of 3) and converted to a total percentage correct score. Participants received an incorrect score if they failed to shoot during a shoot scenario or shot their firearm during a no-shoot scenario. As reported in the original study, no order effects were observed between scenarios at each time point [9].

Subjective SA—Each scenario contained several cues, objects, and behavioural indications by the suspect that officers should use to inform their lethal force DM. Instructors noted if the officer perceived and responded to specific cues that may pose a potential threat (e.g., moving a baseball bat that could be used as a potential weapon to a more secure location) and also further questioned officers during post-scenario debriefings. SA scores were assigned at the end of each scenario by the same instructor who scored their DM on a scale from “Unacceptable” (1) to “Exceptional” (5). 

Each evaluation time point had three SA scores (i.e., one for each scenario), with exception of the post-training evaluation that was assigned one overall SA score. For each evaluation, SA scores for each scenario were summed (out of 15) and converted into a total percentage correct score. SA scores did not significantly differ between scenarios except at 12-month follow-up, with scenarios 1 (M = 3.39) and 2 (M = 4.36) significantly different from each other and lower than scenario 3 (M = 4.43, *p_Bonf_* < 0.004).

### 2.5. Data Analyses

Given that objective DM and subjective SA measures were obtained on different rating scales, they cannot be directly compared. Instead, a series of planned comparisons were used to examine longitudinal patterns of change for each dependent variable. Shapiro–Wilk tests revealed that normality assumptions were violated for lethal force DM scores across all evaluation time points and for SA scores at post-training and 18-month evaluation time points (*p*s < 0.05), requiring non-parametric methods for further analyses. Since there were unequal sample sizes across evaluation time points, we did not run an omnibus Friedman test. Instead, a series of Wilcoxon Signed Ranks tests were run for all pairwise comparisons of evaluation time points, with significance levels adjusted using the Bonferroni correction for multiple comparisons (α = 0.05/10 = 0.005 per dependent variable). All data analyses were completed on SPSS (Version 27, IBM Corp. Armonk, NY, USA).

## 3. Results

### 3.1. Lethal Force Decision Making (DM)

**Figure 1:**
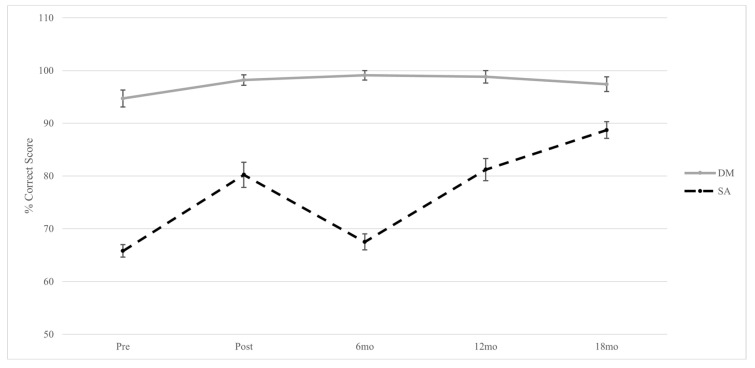
Longitudinal trends of police situation awareness (SA) and lethal force decision making (DM). For lethal force DM, scores remained relatively high and stable with a slight increase from pre-training to 6-month follow-up, and a slight decrease from 6- to 18-month follow-up. For SA, scores significantly increased from pre- to post-training, significantly decreased from post-training to 6-month follow-up, and significantly increased at 12- and 18-month follow-ups. Error bars represent standard error of the mean.

As reported in [9], evaluation scores for lethal force DM did not significantly change across evaluation time points (see Figure 1). Overall, officers made very few lethal force errors as reflected by percent correct scores that exceeded pre-training levels (94.7%) at all subsequent time points (see Table 1). Officers’ lethal force DM improved immediately post-training (98.2%) and was maintained at 6- (99.1%) and 12-month (98.8%) follow-up evaluations, followed by a decrease in DM at the 18-month (97.4%) evaluation. However, none of the pairwise changes to lethal force DM scores were statistically significant at adjusted or unadjusted levels (*p*s > 0.05).

### 3.2. Situation Awareness (SA)

Evaluation scores for SA displayed significant changes across time points (see Figure 1). Pre-training and 6-month follow-up scores (65.8% and 67.5% respectively) were significantly lower than at post- (80.2%), 12- (81.2%), and 18-month (88.7%) follow-up evaluations (see Table 1 and Table 2). 

### 3.3. Trends in SA and Lethal Force DM Evaluation Scores

Several observable differences are apparent when examining longitudinal trends in police UOF performance as operationalised by objective lethal force DM and subjective SA scores (Figure 1). Lethal force DM is relatively stable, with an improvement from pre- to post-training, maintaining a high percent correct score at 6-months follow-up and slightly decreasing at 12- and 18-month follow-ups. DM scores also reflect an extremely high level of competence that exceeds 94% at all time points. In contrast, SA scores displayed more variation between 65% and 89% across evaluation time points. SA scores improved pre- to post-training, decreased at the 6-month follow-up, and increased at 12- and 18-month follow-up evaluations at corrected levels of significance.

Of note, with exception to pre- and post-training scores, changes to SA and DM scores were in opposite directions for the remainder of the longitudinal study; specifically, DM improved from post-training to 6-month follow-up and decreased after 6 months, while SA decreased from post-training to 6-month follow-up and increased after 6 months.

## 4. Discussion

The sample data considered in the current communication reveals apparent differences in how UOF performance is represented by objective and subjective evaluation measures within a sample of active-duty police officers over 18 months. Objective lethal force DM scores were relatively high (>94%) and consistent over time. Subjective SA scores reflected relatively lower levels of competency (65–89%), fluctuated significantly between evaluations, and changes were often in opposite directions than changes to lethal force DM. The data demonstrate that different conclusions about competency could possibly be drawn if only one of these evaluation methods was relied upon to indicate overall proficiency in UOF DM within the current sample of police officers. Further, these data illuminate several important practical and professional implications related to police UOF evaluation, training, and qualification.

### 4.1. Operationalising Competency in Police UOF for the Purpose of Evaluation

Defining and evaluating distinct UOF behaviours and skills is difficult, as they are highly nuanced, complex, and overlap conceptually. Perceptual and cognitive skills such as SA directly inform DM related to the selection of appropriate tactics and UOF behaviours, which themselves are inconsistently defined [4]. As reflected in the current data sample (Figure 1), it is also possible to score well in one outcome but poorly in another, and competency in any individual skill may be lost and regained over the course of a single scenario or across an 18-month period. Therefore, it is important for police instructors and organisations to consider how UOF competencies are operationalised and how to design scenarios to elicit them for the purpose of evaluation and training [21,22]. 

Each scenario an officer encounters requires multiple decisions, especially those that require fast responses to potentially violent or lethal outcomes. For example, consider an officer that needs to search multiple rooms before encountering an armed and hostile suspect. The officer is faced with multiple decisions related to lethal force DM: Should I move to a better location? Should I use my duty weapon or another option? Both of these decisions involve a cognitive *process* (including SA) that is more difficult to evaluate than the final outcome. Objective measures are useful in time-limited contexts where instructors can easily score numerous officers on behaviours that are unambiguous and directly observable (e.g., shooting the armed hostile suspect). However, there should be a high degree of confidence that outcomes accurately reflect psychological and behavioural UOF competencies rather than psychometric properties of the measurement tool itself. As reported in the original study [9], within-subject changes to lethal force DM were non-significant (Figure 1). This may be due to ‘true’ low variability and high competence in directly observable lethal force DM, or due to limited variability in the measure itself, which was operationalised as a binary score rather than a broader range Likert scale such as SA.

Subjective measures provide more detailed information about the *process* by which officers make UOF decisions as well as the *extent* of an officer’s competence, both of which are essential in evaluating the totality of an officer’s skills. Similar to Koedijk et al. [12], investigators in The Netherlands [10] and United States [23,24] have taken a systematic approach to developing validated subjective rating scales for arrest and self-defense behaviours and “objective interval-level metrics” for post hoc scoring of officer UOF and de-escalation performance in real-world incidents, respectively. Both investigators first identify specific behaviours of interest through intensive consultation with police experts and define observable features (or “items”) for optimal and sub-optimal performance. Then, items are rated by a large sample of officers to establish inter-rater reliability for a validated performance scale. Developing validated evaluation measures require significant resources, and while they are still subject to individual interpretation and potential bias, such measures are a significant improvement in standardised practice and raise confidence in the validity of performance outcomes. 

Subjective scalar instructor ratings may be influenced by their personal experiences (i.e., in instruction, training, or field settings), relationships with trainees, or individual factors such as fatigue, observer drift, or perceptual biases (i.e., not observing a behaviour that was performed) [25]. Interpretation skills can be improved through ‘train the trainer’ programs that strengthen evaluators’ abilities in observing, recognising, and interpreting behaviours of interest through effective pedagogical strategies [7,26]. Subjective rating scales are also prone to criterion error, or variability in how individual instructors interpret the scoring criteria for a given measure [25]. Therefore, subjective evaluation criteria should be assessed for inter-rater reliability as suggested above to ensure that assessments by different instructors produce consistent results. Inter-rater reliability for both objective and subjective measures can be promoted by providing detailed criteria for desirable behaviours or skills (see [10,17]), scale endpoints (i.e., what qualifies performance as ‘unacceptable’ or ‘exceptional’), and/or supplementing subjective ratings with objective measures of behaviour, such as frequency or duration [27]. Consistent with the notion that police UOF performance involves a complex set of behaviours and skills, evaluation should collect a variety of subjective and objective measures to form a composite profile of officer competence.

To evaluate an officer’s unobservable knowledge and skills with as little inference as possible, instructors can utilise effective pedagogical strategies such as debriefing and feedback [7,28]. In the previous example, imagine that the officer correctly shoots the armed suspect but fails to consider important tactics related to SA (e.g., clearing a door and corners of multiple rooms that may have hidden additional target persons). In this case, the objective decision to use lethal force is correct but is based on poor SA, which may account for observed discrepancies in individual measures of competency in the current sample (Figure 1). Incorrect application of the skills leading up to a lethal UOF may put officers in a position where they are forced to use it and should be considered during evaluation of UOF performance and avoided through training [17]. Intermittent instructor feedback that “pauses” or “freezes” an ongoing scenario affords officers introspective examination of their current SA and DM strategies [16] and a chance to discuss and adjust these strategies with their instructor before proceeding on an incorrect course of action. Even if it results in the use of lethal force, it is critical that instructors (a) provide constructive feedback and debriefs that prevent encoding or reinforcement of incorrect skill application; and (b) design scenarios that afford officers the opportunity to demonstrate their skills and abilities (i.e., avoid “no-win” scenarios that ambush officers) [7]. These pedagogical approaches will provide valuable learning opportunities while also reducing the likelihood of “training scars” and subsequent OSIs from potentially traumatic occupational exposures [3]. 

The definitions of SA and DM in the current sample data are not the only ways of measuring these constructs or of operationalising UOF performance more generally (i.e., tactics, motor skills). Regardless of whether objective or subjective evaluation measures are used, the purpose of UOF evaluations is to establish competency in the most important skills that officers will require in the field. Therefore, evaluation tasks and scenarios need to be carefully designed to elicit these behaviours. Applied police research suggests that existing UOF practices (i.e., training and evaluation exercises, scenarios, and tasks) do not always translate to operational performance [29]. Indeed, the current lethal force DM performance scores exceeding 94% across all time points (Figure 1) stands in stark contrast to real-world estimates of police shooting accuracy between 22 and 52% [30,31]. Considering that all officers in the current sample data were deemed fit for duty by their agency at the time of each evaluation, the discrepancy between competence in operational and evaluative contexts needs to be reconciled. Several recent commentaries [21,22,32,33] highlight the importance of developing representative tasks that promote fidelity between training and operational contexts. In fact, O’Neill et al. [34] caution that if UOF skills are not generalised to on-duty performance, “officers might be more likely to rely on tools such as chemical spray, conducted-energy devices, or firearms” (p. 366). Combined with increased training frequency to deliberately practice essential UOF skills [35], industry-wide standardisation of police UOF training and evaluation can contribute to improved safety and performance in the field [4,26]. 

### 4.2. Professional and Organisational Considerations for UOF Evaluation

Current assessment of psychological competence in police is typically completed as part of pre-employment assessments [13], while behavioural competencies are rigorously evaluated during basic training. However, training and evaluation standards and practices vary greatly across jurisdictions for all stages of employment [4,26], leaving the definition and evaluation of UOF performance metrics to the discretion of individual services and/or instructors [36]. Thus, UOF evaluation standards are directly informed by existing organisational policies [5]. In the province of Ontario, where the current sample was employed, sworn officers complete annual UOF and firearms evaluations to requalify as ‘fit for duty’. Annual recertification assessments include static target shooting at their agency firing range and a series of UOF scenarios designed to test de-escalation, SA, and lethal force DM competency [2]. Only recently did qualification standards for UOF instructors in Finland require demonstrated competency in tactics, physical UOF techniques, and instruction in addition to weapons handling and safety [7]. Patterns of UOF performance revealed by the current sample data support the importance of repeated longitudinal evaluations of multiple competencies as in [12,14,17], such that consideration of either lethal force DM or SA would indicate discrepant levels of officer competence, especially at the 6-month evaluation (Figure 1).

The current sample data also address some of the practical and organisational concerns above by evaluating police UOF performance during high-fidelity scenarios that meet agency recertification standards. Officers showed high proficiency in lethal force DM that was stable over time and consistently “average” SA, without extremely high or low ratings. While these are desirable findings from an organisational standpoint, they raise a significant question that impacts occupational and public safety: were the scenarios challenging enough? Given a lack of detailed evaluation criteria for SA as in Huhta et al. [17] and a simple ‘yes/no’ binary outcome for lethal force DM, police UOF rating scales may reflect “smile sheets” or evaluations that are generally favourable but uninformative [37]. Organisational influence on the implementation of certain UOF practices over others may encourage “passing” officers because it reduces the burden of remedial re-training and supports existing policy. However, defending the use of potentially ineffective methods and materials, in the absence of adequate training and evaluation, may undermine the professional development and evaluation of police [38,39]. Therefore, it is critical that police organisations and applied researchers examine the effectiveness and potential implications of current operations to ensure that they reflect evidence-based best practices [5]. 

Due to limited resources such as time, funding, and available instructors, annual evaluations are also typically combined with training, which serves a distinct purpose. In-service training is intended to ‘refresh’ existing skills or update knowledge on changes to policies and practices. In some cases, as seen with the Dutch police, regular training of arrest and self-defence skills require only four to six hours a year [40]. A large body of evidence shows significant deficits in police learning, memory, and UOF performance (e.g., verbal de-escalation skills) under high-stress training conditions [41]; for reviews, see [4,42,43]. Stress-induced deficits may be compounded by evaluative stress, such that failing to adequately perform a new or ‘refreshed’ skill will have direct professional implications for the officer (i.e., losing ‘fit for duty’ status, doing remedial training). Therefore, separating training and evaluation wherever possible would improve skilled performance while also promoting skill retention, and has been recommended elsewhere [4,7,44].

### 4.3. Limitations

The sample data presented in this paper were obtained as part of a research study [9], not an agency’s requalification data. As is common in research studies, there were a limited number of participants, behavioural measures, and competencies collected, which was due to logistical constraints (i.e., timing for concurrent training, evaluation, and participation in an external research study). Evaluation measurement scales and criteria, scenario design, and participant scoring were established by independent UOF instructors in line with, and expanding upon agency standards (i.e., SA scores were not in use by the agency at the time of the study and were created for research purposes). Each officer was observed and rated by a single instructor, which also precluded inter-rater reliability analyses of SA and DM outcomes [10]. Ideally, the external validity of evaluation measures should be compared to real-world outcomes (e.g., UOF incident reports) in order to verify that they are a true reflection of the officers’ operational field performance. However, lethal force encounters are extremely rare occurrences [45] and determining the frequency and quality of individual officer’s UOF performance from incident reports would require an extensive and targeted investigation [23,24], which was beyond the scope of the original study or current commentary. 

## 5. Conclusions

Operationalising appropriate measures for police UOF evaluations is difficult given: (a) a lack of standard definitions and conceptual overlap in essential UOF skills and behaviours, (b) inconsistent organisational standards and policies for UOF evaluation, training, and professional certification, and (c) a dearth of applied or empirical evidence examining the validity and consistency of objective and subjective UOF performance metrics. Despite these challenges, there is significant practitioner and public interest in improving UOF evaluation and training.

The current commentary exemplified how two outcome measures (situation awareness and lethal force decision-making) raise separate methodological issues during the evaluation of competency in police UOF, which bear significant professional and personal ramifications. The simplicity of objective evaluation measures comes at the cost of probing important judgement and decision-making skills related to UOF. However, subjective measures are more prone to instructor bias and discrepancy due to increased psychological inference. 

Based on the benefits and limitations of each approach, we recommend that a combination of objective and subjective outcome measures be implemented in a systematic fashion at all stages of police UOF training and evaluation (i.e., basic, advanced, in-service). Developing better standards for police UOF evaluation and training begins with clearly establishing what psychological and behavioural competencies are most important for operational field settings. Only then can representative tasks be designed to evaluate an officer’s level of competence through systematic and evidence-based approaches that in turn promote public and occupational safety. 

## Figures and Tables

**Table 1:** Demographics and performance summaries for lethal force decision-making and situation awareness.

Time	*n* (Female)	Age *M* (*SD*)	Years of Experience *M* (*SD*)	DM % Correct *M* (*SD*)	SA % Correct *M* (*SD*)
Pre-training 3 Scenarios, 3 Decisions	57 (7)	32.8 (6.3)	7.2 (5.6)	94.7 (12.3)	65.8 (9.32)
Post-training 1 Scenarios, 3 Decisions	57 (7)	32.8 (6.3)	7.2 (5.6)	98.2 (7.51)	80.2 (17.6)
6 Months 3 Scenarios, 3 Decisions	39 (3)	33.5 (6.9)	7.7 (6.4)	99.1 (5.34)	67.5 (9.45)
12 Months 3 Scenarios, 3 Decisions	28 (3)	33.6 (6.8)	7.7 (7.0)	98.8 (6.3)	81.2 (11.2)
18 Months 3 Scenarios, 3 Decisions	29 (2)	32.3 (6.0)	6.9 (6.6)	97.4 (7.75)	88.7 (8.56)

Means (*M*) and standard deviations (*SD*) for age, years of experience, objective lethal force decision making (DM), and subjective situation awareness (SA) scores are provided at each time point. With exception of the post-training evaluation, all time points had three scenarios with one lethal force decision and one SA score per scenario. Each correct lethal force decision received a correct score of 1 and SA was scored out of 5 (see measures). The post-training evaluation had only one extended scenario with three lethal force decisions and one SA score (total maximum possible score of SA = 5 and lethal force DM = 3). Scores were converted to percentages (out of 100) and averaged across scenarios (or decisions) at each time point.

**Table 2:** Pairwise comparisons of situation awareness scores between evaluation time points.

Time	Post-Training	6-Months	12-Months	18-Months
Pre-training	−4.93 *^,a^	−0.74 ^a^	−3.90 *^,a^	−4.72 *^,a^
Post-training		−4.09 *^,b^	−0.35 ^b^	−1.77 ^a^
6-months			−3.68 *^,a^	−4.30 *^,a^
12-months				−2.72 ^a^

Two-tailed pairwise Wilcoxon signed ranks tests (*z*) for situation awareness scores between each evaluation time point. Available participant data were matched for each pairwise comparison and varied between *n* = 57 (pre- and post-training), and *n* = 28 (12-months). Effect sizes for significant pairs were between 0.9 > *d* > 2.6. ^a^ Based on negative ranks; ^b^ Based on positive ranks; * *p* < 0.005 (Bonferroni-corrected).

## Data Availability

De-identified data supporting the reported results can be requested from the corresponding author.
